# Two's Company, Three's a Crowd? Maternal and Paternal Talk About Their Infant Differs in Associations With Wellbeing, Couple Relationship Quality, and Caregiving Sensitivity

**DOI:** 10.3389/fpsyt.2020.578632

**Published:** 2020-11-19

**Authors:** Sarah Foley, Carolina Álvarez, Jade McCarthy, Claire Hughes

**Affiliations:** Centre for Family Research, University of Cambridge, Cambridge, United Kingdom

**Keywords:** self-focus, sensitivity, fathers, mothers, anxiety, depression

## Abstract

Problems of depression and anxiety are common in early parenthood and adversely affect parenting quality (1). Rumination is closely linked to poor wellbeing (2), suggesting that self-focus may be one mediator of the association between wellbeing and caregiving [e.g., (3)]. Framed within an international study of first-time mothers and fathers (4), the current study included 396 British mothers and fathers (in 198 heterosexual cohabiting couple relationships) of first-born 4-month-old infants. Parents reported on their symptoms of depression, anxiety and satisfaction in their couple relationship. Five-minute speech samples were transcribed and coded for parents' pronoun use (i.e., “I” and either infant- or partner-inclusive use of “We”), whilst observations in the Still-Face paradigm were coded for parental sensitivity to infants' cues. Our first goal was to test whether new parents' self-focus was associated with wellbeing and couple relationship quality. We also examined whether (i) self-focus mediated the expected association between wellbeing and caregiving sensitivity and (ii) couple relationship quality moderated the expected association between self-focus and caregiver sensitivity. Finally, we compared results for mothers and fathers. Our results illustrate gender-specific associations. First, although mean levels of self-focus and partner-inclusive talk were similar for mothers and fathers, infant-inclusive use of the “we” pronoun was higher in mothers than fathers. Second, self-focus was unrelated to either mothers' or fathers' wellbeing, but was associated with fathers' report of reduced couple relationship quality. In addition, poor perinatal wellbeing was associated with reduced partner-inclusive talk for fathers, but with reduced use of infant-inclusive talk for mothers. Third, mediation models suggest that reduced infant-inclusive talk underpins the association between poor wellbeing and reduced sensitivity in mothers, but not fathers. Fourth, in the context of good couple relationship quality, mothers' elevated partner-inclusive talk was associated with reduced caregiving sensitivity. These findings are discussed in terms of their implications for interventions to support new mothers and fathers, who may benefit from distinct strategies to foster attention to their developing infant.

## Introduction

Becoming a parent is an exciting but challenging time that brings major changes in lifestyle, identity, physical, and mental health (5–7). Approximately one in five new mothers experience serious and persistent symptoms of postnatal depression [PND; (6, 8)] or anxiety (9). Although often overlooked by health professionals, fathers are almost as likely as mothers to develop symptoms of depression and anxiety in the perinatal period (10). Moreover, the past few decades have seen a steady increase in fathers' involvement in caregiving (11), underscoring the importance of including fathers within research on early caregiving (12).

Adopting this approach, Hughes et al. (7) tracked an international (UK, USA, Netherlands) sample of 876 new parents (438 heterosexual couples expecting their first child) from the last trimester of pregnancy to the children's second birthday. Their latent variable analyses demonstrated conceptual equivalence and substantial within-couple concordance in mothers' and fathers' self-reported scores for depression and anxiety. However, their results also showed gender-specific mean wellbeing trajectories (stable for mothers, increased problems over time for fathers). Likewise, key sources of social support associated with improved wellbeing were also gender-specific: friends for mothers, family for fathers. This mixed pattern of results raises questions regarding similarities and contrasts in the cognitive and within-family interpersonal correlates of mothers' and fathers' perinatal wellbeing. To address this question, the current study is focused on detailed data gathered at 4-months from the UK parents and builds on two distinct research traditions.

First, studies framed by cognitive models of depression support the view that increased self-focus—the tendency to consistently focus and assess oneself—contributes to the onset and maintenance of negative affect (13) and may also mediate the impact of depression upon early caregiving. Evidence to support this view comes from an observational study of 54 mothers with 6-month-old infants (3). However, these findings have yet to replicated in larger samples and it is not yet clear whether the conclusions can be extrapolated to fathers. Highlighting the importance of this omission, a second strand of research has demonstrated close links between wellbeing and couple relationship satisfaction [e.g., (14)]. Moreover, each of these constructs show a notable dip following the transition to parenthood (15, 16).

Each of the above traditions can be encompassed within family systems theory (17, 18). One key tenet stemming from this model is the *spillover hypothesis*, which posits that variation in couple relationship quality contributes to variation in the quality of parent-child interactions (19). Numerous studies have documented links between couple relationship quality and self-focus [e.g., (20)], but much less is known about their independence and interplay as predictors of caregiving. By adopting a couples design the current study addressed this gap by examining the associations between self-focus, wellbeing, couple relationship quality and caregiving sensitivity in first-time mothers and fathers.

## Links Between Self-Focus, Perinatal Wellbeing, and Couple Relationship Quality in Mothers and Fathers

Depression has been linked to maladaptive cognitive styles, including increased self-focus (21–23). Sakamoto (24) argued that self-focus contributes to both the onset and maintenance of depression, for example by exacerbating initial response to a negative life event and strengthening negative models of the self that lead to depressed mood (25).

Use of the first-person singular pronoun “I” is a simple, objective and unobtrusive index of individual's attention to the self (26), with classic experimental support [e.g., greater use first-person singular pronouns by individuals who complete a test when they are sat in front of a mirror; (27)]. Two lines of evidence support the construct validity of this measure. First, findings from a meta-analytic review showed a small but consistent positive association: *r* = 0.13 between use of “I” and self-reported levels of depressive symptoms (28). This finding is in line with results from an earlier meta-analysis of 226 studies that found a stronger association between negative affect (i.e., depression, anxiety) and self-focus in clinical than community samples (29). Second, Tackman et al. (30) pooled data from six labs in two countries (USA and Germany) to demonstrate that the frequency of adults' use of “I” shows a small but consistent association: *r* = 0.10 with general distress. Extending this empirical base to include first-time parents, we compared links between self-focus (assessed via the use of “I” in speech samples) and poor perinatal wellbeing in new mothers and new fathers.

Experimental studies of relationship quality have demonstrated that the use of ‘we’ rather than ‘you and I’ leads to heightened perceptions of real and fictitious relationships (31). Similarly, Seider et al. (32) observed couples for 15-min in conflict conversation and found that greater use of “we” was associated with more expressions of positive emotions as well as reduced cardiovascular arousal and expressions of negative emotion. In contrast, frequent use of “I” or “you” was associated with increased displays of negative affect and reduced marital satisfaction.

Two recent expressive writing studies also indicate that pronoun use reflects (and perhaps even contributes to) relationship quality. Robinson et al. (33) gave an expressive writing task to 88 undergraduate students whose partners were later invited to rate couple closeness; this multi-method multi-informant approach showed a small but positive association between use of the first-person plural “we” and partners' ratings of closeness. Unfortunately, a gender imbalance in the study sample (64% women) precluded any comparison of responses from men and women. However, findings from the first expressive writing study to involve married heterosexual couples (*N* = 78, mean age = 40 years for men, 38 years for women) indicate that the use of the plural pronoun “we” is associated with reports of marital satisfaction from women: *r* = 0.26 but not men: *r* = 0.05 (34).

This asymmetry is interesting and potentially relevant to the current study's focus upon early parenthood—a period that is associated with major shifts in the dynamics of family relationships. For most families, the burden of childcare in the early months falls upon mothers (35). Given this asymmetry, the first aim of this study was to test whether self-focus show similar links with poor perinatal wellbeing and couple relationship satisfaction in new mothers and fathers. The findings reported by Allgood et al. (34) suggest the association between couple satisfaction and use of the first-person plural pronoun “we” is likely to be stronger for women than for men.

## Does Self-Focus Mediate the Impact of Perinatal Wellbeing on Caregiving Sensitivity for Both Mothers and Fathers?

We now turn to the second question of whether self-focus might play a mediating role with regards to the impact of poor perinatal wellbeing on parenting behavior. This proposal is framed by theoretical accounts of how parental cognitions influence parental behavior (36, 37), as well as by attachment theory, which highlights parental awareness and interpretation of infant cues as a key foundation for maternal sensitivity (38). From each of these perspectives, self-focus is viewed as constraining new parents' ability to tune into their infants' cues (39, 40).

Experimental work priming rumination provides support for the hypothesized mediating effect of self-focus in the association between wellbeing and caregiving sensitivity (41). In this study 253 mothers with 10-month-old infants [including 90 mothers with generalized anxiety disorder (GAD) and 57 with major depressive disorder (MDD)] were given either neutral or worry/rumination primes in order to examine the impact of self-focus on mothers' thoughts and mother-infant interaction quality. Compared with neutral primes, worry/rumination primes: (i) induced more negative thoughts and self-focus in the sample overall; and (ii) reduced responsiveness to infant vocalizations in mothers with GAD/MDD. DeJong, Fox and Stein (42) argue that these findings add support for their cognitive model of the impact of depression in parenting. Specifically, they propose that depression leads to negative cognitive biases and poor cognitive control, which contribute to rumination that in turn results in delayed or inaccurate responses to infants' cues.

Support for this model comes from a multi-method study of 54 mothers with 6-month-old infants (3). Specifically, this study showed that negative association between self-reported depressive symptoms and researchers' observational ratings of mothers' warmth toward their infant was mediated by variation in mothers' self-focus. In both the study by Humphreys et al. (3), and related studies (33, 34), self-focused was evaluated by coding the relative frequency of “I” and “we” terms from speech samples using the software package “Linguistic Investigation of Word Counts” [LIWC; (43)]. At first glance, automation offers a potentially valuable solution to the time-demands associated with observational research. However, a closer look reveals several possible problems. For example, it is quite common for English-speaking parents to use the second person pronoun “You” to refer to themselves (e.g., “*You come home and you're dog-tired, but you know you need to make an effort for your baby*”). Likewise, in two-parent households the plural first-person pronoun “We” might be used to refer to parent and infant—as assumed by Humphreys et al. (3), but might equally be used to refer to parent and partner; as in the studies by Allgood et al. (34) and by Robinson et al. (33). This point has particular force for first-time parents, as the transition to parenthood necessarily leads to a re-negotiation of relationships as “two” become “three.” Unfortunately, LIWC is not sufficiently sophisticated to distinguish between parents' use of partner-inclusive vs. infant-inclusive first-person plural pronouns (“we” can also be used to refer to the whole family unit, but this usage is, in our experience, much less common).

Furthermore, in keeping with a general propensity to overlook fathers, Humphreys et al. (3) only included mothers in their study. In an exceptional study involving mothers and fathers, Branger et al. (44) found no effect of parent gender on mean levels of caregiving sensitivity observed in routine settings (e.g., lap-play) with 4-month-old infants. However, this study did not explore links between caregiving and either parental wellbeing, couple relationship satisfaction or self-focus. To address these gaps, the second aim of the current study was therefore to assess whether the mediating role of self-focus in the association between low parental wellbeing and poor caregiving sensitivity applies equally to mothers and fathers.

## Interplay Between Self-Focus and Relationship Quality as Predictors of Sensitivity

Becoming a parent involves learning how to interact with one's partner as co-parents, as well as developing a new relationship with the infant (45). Thus, researchers should involve family units rather than individuals and include measures of couple relationship quality alongside individual measures (wellbeing, self-focus) and assessments of parent-infant interactions.

Illustrating this approach, Galdiolo et al. (46) examined the association between use of “we” vs. “I” during a structured conversation about plans for raising their child and the observed quality of structured triadic family interactions (i.e., mother-father-child). In their sample of 47 heterosexual couples with 15-month olds, increased ‘we-ness’ was associated with higher ratings of warmth, whilst greater self-focus was associated with reduced inclusion and validation of one's partner during the interaction. Extrapolating from these findings and the wider literature on couple satisfaction (47), frequent use of infant-inclusive first-person plural pronouns is likely to index perceived closeness to the infant and so variation in the frequency of use of this form of “we” is expected to show a positive association with caregiving sensitivity.

However, family dynamics can be complicated: family systems theory also includes a “compensation” hypothesis that, for example, caregivers might devote extra attention to their infant to compensate for an unfulfilling partner relationship [e.g., (48)]. This possibility is captured by the adage “Two's company, three's a crowd” included in the title for this paper. The third aim of this study was to test whether difficulties in the couple relationship amplify the impact of self-focus on caregiving sensitivity. We hypothesized that this moderation effect would be especially clear for fathers.

## Summary of Main Aims

The current study of 396 first-time mothers and fathers (in 198 heterosexual cohabiting couple relationships) assessed the extent to which new mothers and fathers show common or distinct patterns of association between perinatal wellbeing, self-focus, relationship quality, and observed caregiving sensitivity at 4-months postpartum. To summarize, our study was guided by three research questions:

- Are links between self-focus, poor perinatal wellbeing and couple relationship quality similar for new mothers and fathers?
- Does self-focus play a similar mediating role in the association between perinatal wellbeing problems and caregiving sensitivity for mothers and fathers?
- Does self-focus show an increased salience for caregiving sensitivity in the context of either mothers' or fathers' reduced couple relationship satisfaction?

Overall, we expected to see more similarities than differences between new mothers and fathers in terms of the nature but not necessarily the magnitude of the associations between constructs. First, we hypothesized that the association between new mothers' and fathers' self-focus (assessed via the use of “I” in speech samples) would be of similar strength for perinatal distress and couple relationship quality. Second, for both mothers and fathers, we expected self-focus to underpin the association between distress and reduced sensitivity to infants' cues. Finally, we expected that couple relationship problems would amplify the impact of self-focus on caregiving sensitivity in fathers more than mothers.

## Methods

### Participants

This study reports on the UK-arm of an international prospective study of first-time parents (study name blinded) which sought to investigate the associations between parent wellbeing, parenting behavior and children's self-regulation in the first two years of life. We recruited 221 first-time parent families to the UK-arm of (study name) from antenatal clinics in the East of England. To be eligible participants had to: (1) be first-time parents, (2) expecting delivery of a healthy singleton baby, (3) planning to speak English as a primary language with their child and (4) have no history of severe mental illness (e.g., psychosis) or substance misuse (note this was self-reported by parents and verified by a researcher during the parent interview). Five families were not eligible for follow-up when the infants were 4 months old due to birth complications or having left the country. Of the remaining 216 families, 18 families withdrew and 198 (92% retention rate; Mother *M*age = 31.62, *SD* = 3.86; Father *M*age = 33.36, *SD* = 4.42) agreed to a home visit when their infants (108 boys, 90 girls) were 4 months old, M_Age_= 4.12 months, SD = 0.40 months, range: 2.97 – 5.63 months. All parents were cohabiting, the majority of the sample were highly educated (84.7% of mothers and 77% of fathers had an undergraduate or higher degree), a minority of parents were from ethnic minority backgrounds (9% of mothers and 5% of fathers).

### Procedure

The National Health Service (NHS UK) Research Ethics Committee (name blinded) approved the study protocol (ref number blinded). Parents provided informed consent to be interviewed in the third trimester and at 4-months post-birth and also completed online questionnaires about their wellbeing, couple relationship and family background. At 4 months, parent wellbeing was assessed via online questionnaires. Pairs of researchers conducted two separate home visits to each family, enabling the Still-Face paradigm to be administered twice (counterbalanced once with mother and once with father) without causing undue distress to the infant. Each parent was also invited independently to talk for five minutes about their infant, using the five-minute speech sample paradigm. Parental sensitivity was coded from observations of the Still-Face Paradigm (49) and parental pronoun use was coded from the transcripts of the five-minute speech sample (50).

### Measures

#### Perinatal Wellbeing

Mothers' and fathers' symptoms of anxiety and depression were assessed via the 12-item General Health Questionnaire [GHQ12; (51)], the 20-item Center for Epidemiological Studies Depression Scale [CESD20; (52)], and the six-item State-Trait Anxiety Inventory [STAI; (53)]. Descriptive statistics for these questionnaires are presented in Table 1. A latent factor score was created and used in analyses, whereby a high score was indicative of poorer perinatal wellbeing [for further details regarding the measurement invariance of this measure please see, (7)].

**Table 1:** Descriptive Statistics and Reliability Information for 4-month Maternal and Paternal Questionnaires and Observation Measures.

**4-month measures**	**Mother (*****N*** **=** **198)**			**Father (*****N*** **=** **198)**		
	***M***	***SD***	**α**	***M***	***SD***	**α**
1. CESD	8.75	6.93	0.87	9.12	6.91	0.87
2. GHQ	1.57	2.19	0.81	2.26	1.74	0.81
3. STAI	10.21	2.88	0.77	11.15	3.13	0.81
4. CSI	69.27	9.60	0.96	68.24	11.49	0.96
5. CTS (reversed)	30.24	2.21	0.64	30.12	2.19	0.64
6. Observed sensitivity	1.71	0.80	-	1.43	0.74	-

*CESD, Center for Epidemiological Studies Depression Scale; GHQ, General Health Questionnaire; STAI, State-Trait Anxiety Inventory; CSI, Couple Satisfaction Index; CTS, Conflict Tactics Scale (reverse scored); Observed sensitivity, adapted version of the global sensitivity rating scales; ICC, 0.82*.

#### Couple Relationship Quality

Mothers and fathers reported on their happiness and satisfaction in the couple relationship using the 16-item Couple Satisfaction Index (54). Parents also reported on the frequency with which they engaged/experienced negative interactions with their partner using the 6-item Conflict Tactics Scale (55). Parents scores on negative items were reverse coded so that high scores reflected low levels of conflict. Descriptive statistics for these questionnaires are presented in Table 1. A latent factor score was created and used in analyses, whereby a high score was indicative of greater relationship quality [for further details please see (4)].

#### Pronoun Use

Both parents provided a five-minute speech sample (FMSS) describing their infant and their relationship with their child (50). Specifically, they were instructed: “*I'd like to hear your thoughts and feelings about your baby, in your own words and without my interrupting with any questions or comments. When I ask you to begin I'd like you to speak for 5 minutes, telling me what kind of a person your baby is and how the two of you get along together.”* These speech samples were audio-recorded, transcribed verbatim and pronoun use was coded in a two-step process. First, we manually analyzed each transcript to distinguish between parents' use of “I” to refer to “I the child,” “you” to refer to themselves as the parent and “we” meaning either “I and Baby,” “I and Father/Mother,” or “I, Baby and Father/Mother.” Second, [and following the approach employed by Humphreys et al. (3)], to count the direct use of the pronoun “I,” all the transcripts were read into the text analysis program Linguistic Inquiry and Word Count [LIWC; (43)]. From this basis, we then combined the manual and LIWC count for “I,” and subtracted references to “I” which referred to their baby (i.e., exceptions that the automated software could not detect). As a result, we had three scores reflecting total self-focus, infant-inclusive and partner-inclusive talk. Examples of talk coded for pronoun use are given in Table 1.

#### Observed Parent Sensitivity

During the two home visits each parent completed the Still-Face paradigm with their infant. The five-minute still-face paradigm consists of three episodes; the baseline where the parent and infant interact as normal, the still-face where the parent ceases interaction and adopts a neutral face, and the reunion where normal face-to-face interaction is resumed (49). Sensitivity, based on gaze direction, vocalization, and verbalization, was coded using an adapted version of the 4-point global sensitivity rating scales (56, 57). Reliability was established on 20% of the samples, sensitivity ICC = 0.82, and descriptive statistics are presented in Table 1.

### Analysis Plan

Pearson's correlations were used to examine the association between parent pronoun use, perinatal wellbeing, couple relationship quality, and sensitivity. We used a regression model to test whether difficulties in the couple relationship moderated the impact of self-focus on mothers' and fathers' sensitivity. Specifically, we added three terms to index the interaction between couple relationship quality and each talk measure (i.e., self-focus talk, partner-inclusive talk, and infant-inclusive talk). We mean-centered both of the independent variables prior to calculating the interaction term and ran models separately for mothers and fathers, controlling for total word count and the length of the couple relationship. Following this we tested whether poor perinatal wellbeing increases the likelihood of being self-focused during infancy, which in turn reduces parents' sensitivity. A model to test for this indirect effect of depression on sensitivity via talk was specified using bootstrapping procedures with 10,000 bootstrap samples (58). All of the models were run using M*plus* [Version 8; (59)] and model fit was assessed using Brown's (60) recommended criteria: non-significant chi-square, root mean square error of approximation (RMSEA) ≤ 0.06, comparative fit index (CFI) ≥ 0.90 and Tucker-Lewis Index (TLI) ≥ 0.90. Due to the non-normal distribution of the talk scores we used the robust maximum likelihood estimator with robust standard errors (MLR). Five-minute speech samples were missing from seven mothers and four fathers and so we adopted a full information approach to data analysis using the sample of 198 families. This approach is suitable for regression models and produces less biased estimates than traditional missing data handling procedures (61).

## Results

### Preliminary Analyses: Mothers' and Fathers' Self-focus

Table 2 presents examples of self-focus, infant-inclusive and partner-inclusive talk coded from parents' speech samples. As described above, we supplemented automated coding of the speech samples using the LIWC software (43) with manual coding to capture parents' use of the second person pronoun “You” to refer to themselves. As shown in Table 3, which presents the means (*M*), standard deviations (*SD*) and ranges of the pronoun use variables, the enhanced self-focus measure captured significantly more of parents' references to the self than the LIWC software for both mothers, *t*_(191)_ = 13.94, *p* < 0.001, Cohen's *d* = 0.29, and fathers, *t*_(193)_ = 14.13, *p* < 0.001, Cohen's *d* = 0.29. Thus, this enhanced self-focus measure was used in all subsequent analyses. Table 3 also reports the comparisons between mothers' and fathers' talk. These showed a modest contrast (favoring mothers) in frequency of infant-inclusive pronouns. However, mothers and fathers were similar on all other talk variables.

**Table 2:** Coding of Pronoun Use in Five-Minute Speech Samples.

**Pronouns included in count**	**Examples**	**Context**
“I” to mean “I” “You” to mean “I” “We” to mean “I and Infant” “We” to mean “I and Partner”	“*I always thought”* “*I look forward to”* “*it makes you feel like…”* “*you can sometimes think that…”* “*we get along well”* “*we will read a book before bed”* “*we would like another child”* “*we applaud him”*	Clear reference to self Speaking about own feelings Speaking about own thoughts Describing the relationship specifically between self and child Describing an activity that the parent and child will do together Speaking about self and partner with no reference to the child Speaking about self and partner with reference to the child
**Pronouns excluded from count**	**Examples**	**Context**
“I” to mean “Infant” “You” to mean “Infant” “You” used in a general phrase “You” to mean the interviewer “We” to mean self and interviewer “We” to mean parent and other family members	“*I don”t want to cuddle anymore”* “*I don”t want you I want Mum”* “*you like to play with this toy”* “*you laugh a lot”* “*you know”* “*if you like”* “*you can see”* “*as you already know”* “*how much time do we have left?”* “*like we spoke about earlier”* “*we went to the park”*	Speaking from the perspective of the child rather than the self Clear reference to the child rather than to self or interviewer Linguistic fillers or phrases used in speech with no reference to anyone Directly referring to the interviewer, often in the context of other situations or instances the interviewer is familiar with Speaking about the interview or previous conversations, grouping her/himself with the Interviewer Referring to family members who are separate from mother, father and child

**Table 3:** Means, Standard Deviations, Range, and Comparison between Mothers' and Fathers' Use of Self-Focused, Infant-Inclusive, and Partner-Inclusive Pronouns.

	**Mother (*****N*** **=** **191)**			**Father (*****N*** **=** **194)**			**Comparison**		
**Talk**	***M (SD)***	**Range**	**Skew (*SE*)**	***M (SD)***	**Range**	**Skew (*SE*)**	***t***	***p***	***d***
Self-focus	39.16 (16.42)	12–97	0.86 (0.17)	38.71 (19.17)	4–114	0.55 (0.18)	0.44	0.657	0.03
LIWC Self-focus	34.53 (15.43)	5–88	1.00 (0.18)	33.34 (17.8)	3–99	0.59 (0.18)	0.75	0.454	0.07
Infant-inclusive	4.78 (3.52)	0–16	1.91 (0.17)	4.06 (4.29)	0–22	0.79 (0.18)	2.09	0.038	0.18
Partner-inclusive	5.02 (5.23)	0–36	1.86 (0.17)	5.48 (5.49)	0–32	2.00 (0.18)	−1.03	0.306	0.09
Total talk	670.85 (187.50)	183–1115	0.36 (0.18)	669 (232.16)	148–1497	0.14 (0.17)	−0.27	0.791	0.01

*Self-focus = LIWC plus manual coding of personal pronouns; LIWC self-focus = automated coding of pronoun use*.

### Is Poor Perinatal Wellbeing and Couple Satisfaction Associated With Increased Self-focus in Mothers and Fathers?

For mothers, perinatal wellbeing was unrelated to our index of self-focus, namely the use of the first-person singular pronoun “I” (*r* = −0.04, *p* = 0.582) but weakly negatively associated with use of both infant-inclusive: *r* = −0.12, *p* = 0.097, and partner-inclusive *r* = −0.13, *p* = 0.082, use of the first-person plural pronoun “we” (see Table 4). For fathers, poor perinatal wellbeing was likewise negatively associated with partner-inclusive use of the first-person plural pronoun “we”; *r* = −0.17, *p* < 0.016, but was unrelated to either self-focus, *r* = *0.0*6, *p* = *0.4*31, or infant-inclusive talk, *r* = 0.04, *p* = 0.543. There was no significant between-parent difference in the strength of association between wellbeing and infant-inclusive pronoun use (z = 1.59).

**Table 4:** Correlations between Main Study Measures.

	**Mother**			**Father**		
	**Wellbeing**	**Couple Relationship Quality**	**Observed Sensitivity**	**Wellbeing**	**Couple relationship Quality**	**Observed sensitivity**
Self-focus talk	−0.04	0.01	0.06	0.06	−0.13^+^	0.04
LIWC self-focus talk	−0.07	0.05	0.05	0.09	−0.11	0.04
Partner-inclusive talk	−0.13^+^	−0.03	−0.18^*^	−0.17^*^	0.04	0.01
Infant-inclusive talk	−0.12^+^	−0.04	0.22^**^	0.04	0.06	−0.02
Total talk	0.00	−0.02	0.02	0.00	−0.02	0.04

*Self-Focus = LIWC plus manual coding of personal pronouns; LIWC self-focus = automated coding of pronoun use*.

+ *p < 0.10*;

* *p < 0.05*;

** *p < 0.01*.

For fathers, couple relationship quality was, as expected, negatively related to self-focus, *r* = −0.13, *p* = 0.067, though unrelated to mothers' self-focus, *r* = 0.01, *p* = *0.8*61 (see Table 4). However, both parents' reports of couple relationship quality were unrelated to frequency of either partner-inclusive (mother *r* = −0.03, *p* = 0.639, father *r* = 0.04, *p* = 0.567) or infant-inclusive pronoun use (mother *r* = −0.04, *p* = 0.631, father *r* = 0.06, *p* = 0.442).

### Does Pronoun Use Mediate the Association Between Poor Perinatal Wellbeing and Sensitivity?

Although perinatal wellbeing and sensitivity were not directly related in this sample (see Table 4), “domino” chain reactions mean that direct effects are not necessary to demonstrate mediation (58). Given the positive findings reported by Humphreys et al. (3), we posited that poor perinatal wellbeing increases the likelihood of being self-focused during infancy, which in turn reduces parents' capacity to be sensitive to their infants' cues.

**Figure 1:**
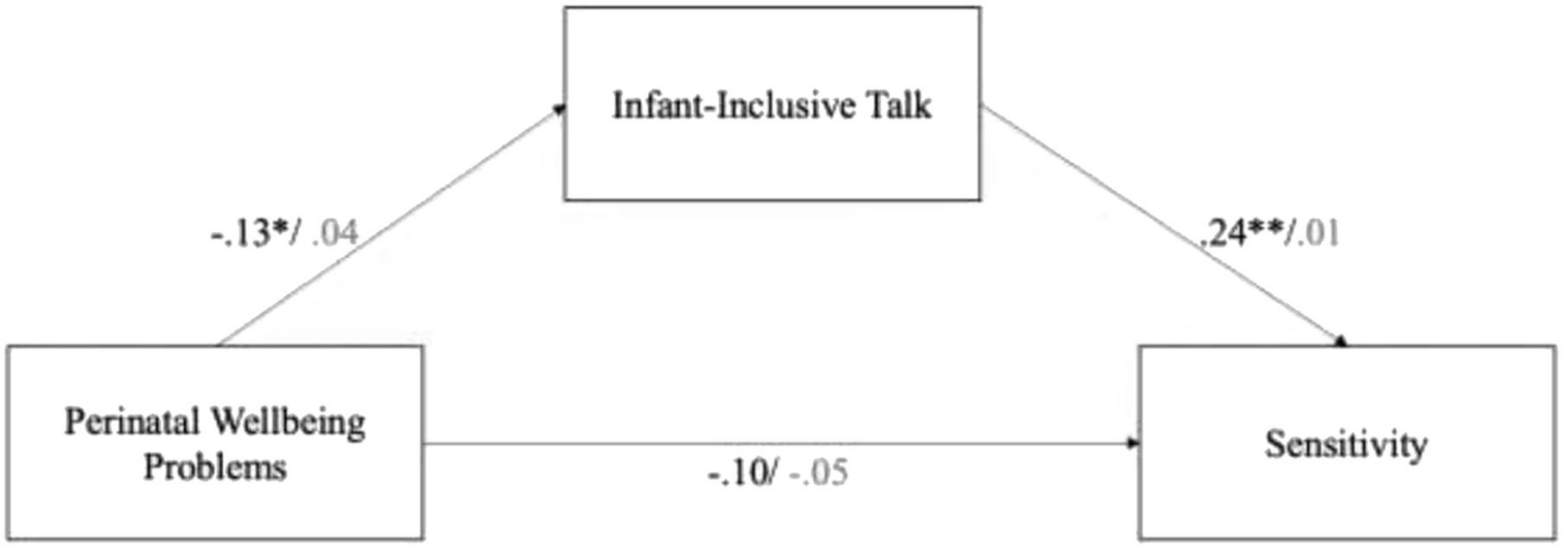
Mediation model, mothers standardized estimates in black/fathers in gray. Model controls for total word count. ^*^p < 0.5, ^**^p < 0.1.

First, we examined maternal sensitivity, with word count as a covariate, and three potential mediators: self-focus, infant-inclusive, and partner-inclusive pronoun use. The *infant-inclusive* model showed good fit, RMSEA = 0.000, 90% CI [0.00, 0.03], CFI = 1.00, TLI = 1.00. The unstandardized estimate of the indirect effect of infant-inclusive talk and 95% confidence intervals with 10,000 bootstrap samples was significant, 0.01 [0.01, 0.03], indicating a modest but indirect effect of poor perinatal wellbeing via infant-inclusive talk on maternal sensitivity (see Figure 1). Both indirect effects for the corresponding mediation models involving self-focus, 0.00 [-0.01, 0.04], and partner-inclusive talk, 0.00 [-0.01, 0.03], included 0 within their 95% confidence intervals indicating a non-significant indirect effect.

Second, we ran the same mediation models for fathers. Unlike for mothers, as illustrated in Figure 1, we found no support for the idea that infant-inclusive talk mediated the association between fathers' perinatal wellbeing and sensitivity,−0.01 [-0.02, 0.01]. We also found no evidence that self-focus,−0.01 [-0.02, 0.03], nor partner-inclusive talk,−0.01 [-0.02, 0.01], mediated the association between fathers' perinatal wellbeing and sensitivity.

### Do Couple Relationship Problems Amplify the Impact of Self-focus on Sensitivity?

**Figure 2:**
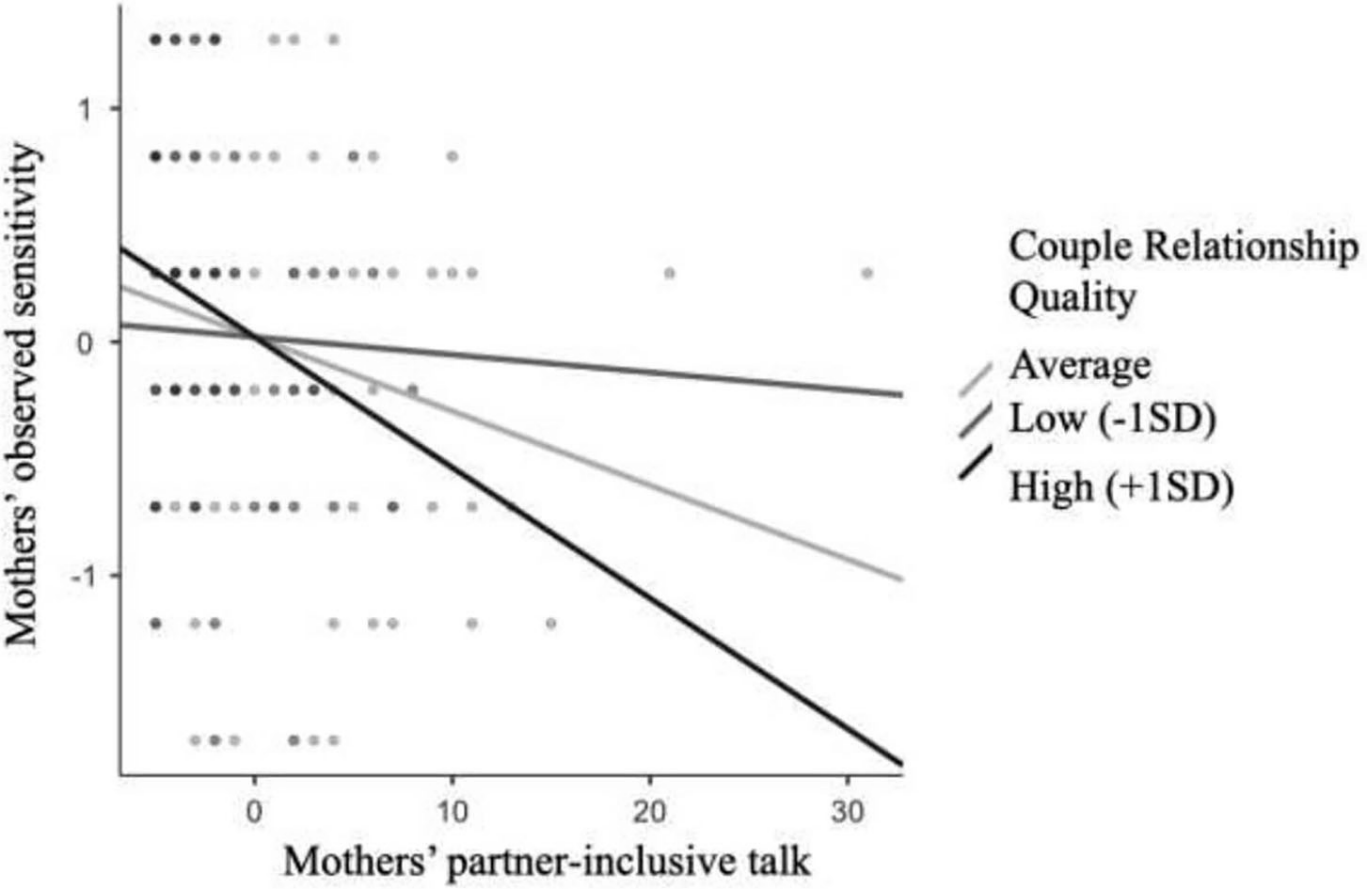
The association between mothers' partner-inclusive talk and mothers' observed sensitivity during the still-face paradigm by couple relationship quality.

We tested the proposed moderation effect of couple relationship quality on the association between pronoun use and parents' sensitivity, controlling for length of relationship. For mothers, one just-identified model provided support for the hypothesized moderation effect, as the interaction term between couple relationship quality and partner-inclusive talk was significant, β = −0.23, SE = 0.08, *Z* = −3.04, *p* = 0.002. We probed this interaction using simple slope analysis. This *post-hoc* exploration involves creating new terms to reflect low (−1SD), average and high (+1SD) values of the interaction terms and calculating the slope of each condition. As illustrated in Figure 2, we found the slopes reflecting interactions between average, *Z* = −2.80, *p* = 0.005, and high, *Z* = −3.34, *p* = 0.005, couple relationship quality and partner-inclusive talk to be significant [note Jamovi (62) was used to illustrate this interaction]. That is, in the context of good couple relationship quality, greater maternal use of partner-inclusive talk was associated with reduced sensitivity to infants' cues during the still-face interaction. However, neither the interaction terms between couple relationship quality and self-focus, β = −0.10, SE = 0.17, *Z* = −0.51, *p* = 0.612, nor infant-inclusive talk, β = −0.01, SE = 0.10, *Z* =−0.08, *p* = 0.935 respectively, were significant.

For fathers, one just-identified model provided support for the hypothesized moderation effect, as the interaction term between couple relationship quality and self-focus talk was significant, β = −0.36, SE = 0.07, *Z* = −2.66, *p* = 0.008. However, following the same simple slope *post-hoc* procedures as outlined above, we found no significant differences between in the slopes reflecting low, average, and high couple relationship quality and self-focus, Z <1.5, *p* > 0.100. In addition, neither the interaction terms between couple relationship quality and partner-inclusive, β = −0.09, SE = 0.17, *Z* = −0.52, *p* = 0.601, nor infant-inclusive talk, β = 0.02, SE = 0.16, *Z* = 0.10, *p* = 0.924 respectively, were significant.

## Discussion

This study involved 396 first-time mothers and fathers (in 198 heterosexual cohabiting couple relationships) who were each asked to talk for 5 min about their relationship with their 4-month-old infant. Automated and manual coding of these speech samples were used to obtain frequencies of first-person singular and plural pronouns, which were used as indicators of self-focus and parent-infant closeness. These two constructs were then examined in relation to self-reported parental wellbeing and couple relationship quality, as well as observational ratings of caregiving sensitivity. Mediation and moderation models were used to elucidate the processes underpinning the impact of postnatal problems of depression and anxiety on caregiving sensitivity in both mothers and fathers.

Our analyses yielded four key findings. First, fathers' self-focus was related to poor couple relationship quality but not perinatal wellbeing. Second, mothers' reduced ability to focus upon her relationship with her infant mediated the impact of postnatal symptoms of depression and anxiety upon caregiving sensitivity. Third, mothers' frequent use of partner-inclusive pronouns was associated with reduced sensitivity to the infant—and this especially clear in the context of a positive partner relationship. This moderation effect supports the adage “Two's company, three's a crowd” —at least for new mothers, who may be experiencing a time of divided loyalties. Fourth, the above findings were largely gender specific, as our sample of first-time fathers of 4-month-old infants did not show significant mediation or moderation effects. Below we discuss these findings in turn.

### Self-focus in New Parents Is Related to Poor Couple Relationship Quality Rather Than to Perinatal Wellbeing

Earlier, we noted that Nilly and Winquist (29) reported a stronger association between self-focus and symptoms of negative affect for clinical samples. In this context, the lack of association between wellbeing and self-focus may reflect the demographically low-risk nature of our sample. However, wellbeing was associated with levels of partner-inclusive talk for both mothers and fathers and also associated with maternal levels of infant-inclusive talk. Two factors indicate that the lack of association between wellbeing and self-focus is unlikely to reflect a floor effect. First, on average, first-person singular pronoun “I” accounted for 5.11% of total talk (*SD* = 1.90%), echoing results from other studies [e.g., (33), *M* = 5.22, *SD* = 2.29%]. Second, this average rate was—for both mothers and fathers—approximately six times higher than for either partner-inclusive or infant-inclusive exemplars of the pronoun “we.” However, these latter measures did show greater variability and so may have been more sensitive to wellbeing-related contrasts in pronoun use. Thus, future studies might benefit by supplementing tallies of the first-person singular pronoun “I” with more nuanced measures of self-focus. For example, Woodruff-Borden et al. (63) found that whilst negative self-focus was positively associated with five different measures of psychological distress, positive self-focus was *negatively* associated with symptoms of depression, anxiety and positively associated with social problem-solving skills.

Alternatively, our study findings may differ from prior studies because all of our participants had newly made the transition to parenthood, a period in which couple relationships show considerable change, with potential consequences for wellbeing. In the current study, fathers' self-focus was inversely related to couple relationship quality. Additional correlational analyses highlighted a negative association between couple relationship quality and poor perinatal wellbeing (*r* = −0.14^*^ for mothers and *r* = −0.33^**^ for fathers). This overlap echoes findings from empirical and intervention research that demonstrate the importance of partner support for mental health across the transition to parenthood (64, 65). Future research examining links between self-focus, mood and couple relationship satisfaction would benefit from including individuals at different stages of parenthood.

### For New Mothers, Reduced Infant Focus Mediates the Impact of Poor Perinatal Wellbeing Upon Caregiving Sensitivity

Our results showed no direct inverse association between caregiving sensitivity and self-focus. However, reduced sensitivity in the Still Face paradigm was related to mothers' infrequent use of infant-inclusive pronouns. Our study therefore provides indirect support for the results reported by Humphreys et al. (3), as well as for reports that maternal depression shows positive associations with rumination [e.g., (66)] and negative associations with other markers of infant focus, including levels of mind-mindedness [e.g., (67, 68)]. These findings converge with DeJong et al. (42) cognitive model that suggests postnatal depression leads to cognitive biases which restrict mothers' cue processing.

That said, it is worth noting that pronoun use provides only one index of self-focus. Meta-analytic studies of the related construct of rumination highlight two dimensions: reflective pondering and brooding [e.g., (69)]. Given this distinction, although pronoun use is a widely used and considered an objective measure of self-focus (70), future replication research might benefit from also including different measures of self-focus, for example sentence completion stems (71).

### Two's Company, but Three's a Crowd: Making Room for Baby

Extending the developmental scope of previous work with toddlers (46), our study of parents with 4-month old infants showed an inverse association between partner-inclusive talk and caregiver sensitivity. Furthermore, unlike Galdiolo et al. (46), our relatively large sample size allowed us to test whether couple relationship quality strengthened the association between parents' self-focus and individual caregiver sensitivity. Drawing on findings that fathers are especially susceptible to spill-over effects (72), we expected this moderation effect to be particularly clear for fathers. Instead, we found that greater partner-inclusive talk was only associated with reduced sensitivity to infants' cues in mothers—and that this inverse association was amplified in the context of high couple relationship satisfaction. As parents were instructed to talk about their thoughts and feelings about their baby and how they were getting along with their baby, frequent reference to participants' marital partners might reflect difficulties in staying focused on the infant during the speech sample task. This difficulty in staying “present” may underpin the association between partner-inclusive talk and reduced caregiving sensitivity.

Symbolic of interdependence, use of the first-personal plural pronoun “we” has been linked with investment within relationships (47) and is seen as a means through which individuals make cognitive room for one another's psychological and emotional needs (73). As such, this moderation effect may actually be driven in the opposite direction. That is, relationships characterized by high levels of satisfaction and low levels of conflict may be a place of solace for new mothers experiencing difficulties in learning how to respond sensitively to their infants' distress cues. Our nuanced findings mirror results from a subsample of 93 families who took part in in-depth recordings of their family-talk environment at 7-months (48). Specifically, compared with mothers of daughters, mothers of sons who reported less satisfaction and more conflict in their relationship with their partner used more infant-directed speech. Taken together, our findings may also suggest a compensation effect, whereby mothers invest more energy into their interactions with their child when their couple relationship is not functioning as well.

### Gender-Specific Links Between Perinatal Wellbeing, Self-focus, Couple Relationship Quality and Caregiving Sensitivity

Our results showed that reduced infant-inclusive talk was associated with symptoms of depression and anxiety in mothers, but not in fathers. That said, the difference in the strength of this association for mothers and fathers was not statistically significant. In contrast, the association between observational ratings of caregiving sensitivity and infant-inclusive talk was significantly stronger in mothers than in fathers.

Interestingly, the rather distinct patterns of results for fathers and mothers in this study contrasts with the broadly similar results for mothers and fathers in other analyses involving the same study sample. These include the finding that mothers and fathers provide conceptually equivalent ratings of their own symptoms of depression and anxiety (7) and do not, on average, differ in mean levels of caregiving sensitivity at 4-months (44). Other analyses from this study sample have also revealed striking between-parent similarities in: (a) the interplay between difficult birth experiences and postnatal wellbeing (7); and (b) associations between prenatal symptoms of anxiety and depression and infant adjustment at 24-months (4). That said, day-long recordings of family talk in a subsample of these families showed more frequent maternal rather than paternal infant-directed speech at 7 months (48). Outside of this sample, there are inconsistent reports of gender-related contrasts in parent sensitivity [e.g., (74, 75)]. Further research, mindful of the oft interconnected nature of parent gender and caregiver role, will be helpful in teasing apart the nature of these differences [c.f., (76, 77)].

However, one plausible explanation of these contrasting results is that the asymmetry in findings for mothers and fathers is restricted to caregiving under stressful conditions. Indeed, it has been noted that the impact of poor maternal wellbeing on mothers' caregiving is more likely to be seen under stressful conditions (42). Furthermore, the Still-Face paradigm is especially suited to assessing parental sensitivity to infant distress, a behavior that is known to be differentially associated with mother-infant and father-infant attachment security (78). This dissonance may reflect the primary caregiver (typically mothers) fulfilling the “safe haven” function of the attachment relationship (i.e., providing comfort when distressed) (79), whilst fathers provide more of a “secure base” for exploration of the environment (80, 81). Consistent with this view, studies have shown that fathers who engaged in greater physical and object stimulation during interactions are more likely to be rated as having secure relationships with their infants (82). If fathers spend the majority of their time with their children in play rather than caregiving activities (83), their capacity to respond to infant distress cues in the stress-provoking still-face paradigm may not capture the salient features of paternal sensitivity that may be comprised by depressive symptoms. Therefore, future investigations of links between wellbeing and parent-child interactions should adopt a differentiated model that encompasses measures of parenting in distinct contexts.

## Implications and Future Directions

Cognitive behavioral therapy (CBT) aims to identify, evaluate and modify negative cognitions and dysfunctional beliefs [e.g., (84)] and is widely used both to treat depression (85) and minimize the negative impact of depression on mothers' parenting and subsequent child outcomes (86). Our mediation findings suggest that targeting mothers' cognitive style may also help reduce the intergenerational transmission of depression in community samples. At the same time, our findings suggest that interventions originating from maternal frameworks may not necessarily simply translate for use with fathers [c.f., (76, 77)].

Another important goal of future research would be to examine the experience and impact of poor perinatal wellbeing in samples from different cultural and ethnic backgrounds, who may differ from Western samples in both caregiving [e.g., (87)] and self-focused cognitions [e.g., (88)]. For example, given that different cultural scripts impact the presentation of depression across cultural contexts (89), it is likely that poor perinatal wellbeing will have a varied impact on parenting between and within cultures.

Other exciting avenues for future research concern examining direct links between parents' self-focus and child outcomes. Two longitudinal studies have reported predictive links between parents' rumination and poor pre-school outcomes; the first demonstrated direct negative effects of fathers' brooding rumination on pre-school emotional symptoms (90), whilst the second reported that rumination mediated the association between maternal depression and maladaptive emotion regulation in pre-school children a year later (91). Testing whether parental self-focus has direct effects on child outcomes will provide important theoretical and practical contributions.

## Strengths and Limitations

Three study limitations deserve note. First, the cross-sectional and correlational nature of our analyses mean that our findings should be interpreted with caution. Longitudinal work is needed to illuminate both the direction and developmental specificity of mechanisms underpinning associations between wellbeing and parenting behavior. Second, our sample reflect a self-selected group of highly educated first-time parents who were willing to opt-in to a project that would involve filmed parent-infant interactions with a child who they had yet to meet. Our findings therefore also require replication in studies involving more diverse samples. Third, while our measure of parents' sensitivity was based on the well-validated still-face paradigm (92), these parent-child observations were originally developed with mothers. As discussed earlier, future research adopting alternative coding schemes will enable us to test the specificity of this mediation model, for example coding sensitive responding during play or cognitive sensitivity may be particularly relevant for fathers (93).

That said, our study did also have a number of strengths. These include the involvement of a relatively large sample size (396 parents), who were assessed using a variety of methods (i.e., questionnaire, interviews, and observations). In addition, multi-measure indexes of couple relationship quality and perinatal wellbeing showed conceptual equivalence for new mothers and fathers, strengthening the reliability of our findings (7). In addition, our supplementary use of manual coding for pronoun use enabled us to examine subtle differences in the referents of parents' narratives, allowing us to distinguish, for example, between partner-inclusive and infant-inclusive plural pronouns—a distinction that may be especially salient for new parents.

## Conclusions

Our study tested the links between self-focus, perinatal wellbeing, and mothers' and fathers' sensitivity to their 4-month old infants' cues. By including fathers as well as mothers and by adopting a fine-grained measure of self- vs. other-focus, we aimed to identify the specificity of the cognitive underpinnings of the well-known link between poor wellbeing and parent-infant interaction quality. Our gender-specific findings highlight the danger of extrapolating from mother-focused models to fathers. First, symptoms of depression and anxiety were related to reduced infant-inclusive talk—but only for mothers. Interestingly, variation in fathers' self-focus appeared related to poor couple relationship quality rather than to perinatal wellbeing. Likewise, our mediation models suggest that reduced infant-inclusive talk underpins the association between symptoms of depression and reduced maternal (but not paternal) sensitivity to infants' cues. Third, frequent partner-inclusive pronoun use was associated with reduced sensitivity to the infant—but again, only in mothers in the context of a positive partner relationship. Our results also highlight the value of recognizing the family context within perinatal mental health research. Future research would benefit from examining the extent to which parent and infant characteristics may moderate the strength of these associations, as well as testing whether these findings replicate across development and different domains of parenting.

## Data Availability Statement

The datasets generated for this study can be found online at the UK Data Service repository: Hughes et al. (94).

## Ethics Statement

The studies involving human participants were reviewed and approved by NHS London Bloomsbury REC. Protocol Reference: 14/LO/1113. Written informed consent to participate in this study was provided by the participants.

## Author Contributions

SF: data collection, data curation, formal analysis, writing–original draft. CÁ: data coding, writing–review and editing. JM: data coding, writing–review and editing. CH: supervision, funding acquisition, methodology, writing–original draft. All authors contributed to the article and approved the submitted version.

## Conflict of Interest

The authors declare that the research was conducted in the absence of any commercial or financial relationships that could be construed as a potential conflict of interest.
